# Tetrandrine reverses drug resistance in isoniazid and ethambutol dual drug-resistant *Mycobacterium tuberculosis* clinical isolates

**DOI:** 10.1186/s12879-015-0905-0

**Published:** 2015-03-25

**Authors:** Zhe Zhang, Jie Yan, Kaijin Xu, Zhongkang Ji, Lanjuan Li

**Affiliations:** Research Center of Infection and Immunity, Department of Microbiology and Parasitology, School of Basic Medical Sciences, School of Medicine, Zhejiang University, Hangzhou, China; State Key Laboratory for Diagnosis and Treatment of Infectious Diseases, Collaborative Innovation Center for Diagnosis and Treatment of Infectious Diseases, the First Affiliated Hospital of School of Medicine, Zhejiang University, 15th floor, Building 6A, No. 79 Qingchun Road, Shangcheng District, Hangzhou, 310003 Zhejiang Province China

**Keywords:** Tetrandrine, Drug resistance, Efflux pump, *Mycobacterium tuberculosis*

## Abstract

**Background:**

Tetrandrine is a natural chemical product purified from *fourstamen stephania* root which recently has been shown to act similarly as synthesized drug efflux pump inhibitor verapamil. The aim of the study is to examine whether tetrandrine could potentiate anti-tubercular drugs to which *Mycobacterium tuberculosis* (MTB) has turned resistant via efflux mechanisms.

**Methods:**

We screened 200 MTB clinical isolates using drug sensitivity test to look for those who have turned resistant to the drugs most probably due to efflux mechanisms. We found 29 isoniazid (INH) and ethambutol (EMB) - dual resistant (IEDR) strains. Then we tested if treatment with tetrandrine or verapamil could reverse drug resistance to INH and/or EMB in IEDR isolates.

**Results:**

There is a parallel resistance among EMB- and INH-resistant strains in the tested clinical isolates. Among INH-resistant strains, 65% was also EMB-resistant. This suggests an involvement of efflux mechanisms which can lead to dual drug resistance in IEDR clinical isolates. Similar to a synthesized efflux pump inhibitor verapamil, tetrandrine treatment together with INH or EMB brought down the MICs from the clinical level of drug resistance to the sensitive range of both drugs. The effective rate reached 82% among IEDR clinical isolates.

**Conclusions:**

Combinational application of tetrandrine with INH or EMB increased drug efficacy. Drugs like tetrandrine may help to reduce drug dosage thus alleviate side effects.

## Background

Due to the emergence of drug-resistance, many anti-tubercular drugs end up not useful anymore [1]. An urgent goal of anti-tubercular drug discovery is to find drugs that can help to overcome drug resistance [1]. Drug resistance in MTB rises mostly from chromosomal mutations. Such mutations were not found in some drug-resistant clinical strains, where efflux mechanisms may be held accountable [1-6]. Efflux pumps can pump out toxic compounds of a variety of structures. This is because efflux pumps have an extremely large central cavity, which allows binding of different ligands using a different subset of pump residues through hydrophobic, aromatic stacking and van der Waals interactions [3,7]. Thus it is easy to see why efflux pumps may mediate simultaneous drug resistance to drugs of various structures.

Tetrandrine (*6,6',7,12-tetramethoxy-2,2'-dimethylberbam*, TET) is a low-toxicity drug extracted from the plant *Stephania tetrandra* S. Moore (Fenfangji) of the menispermaceae. It has been used in the treatment of hypertension, cardiac arrhythmia and angina pectoris in China since the 1950s and few side effects have been noted in the clinical practice [8]. TET has been shown to be a Ca^2+^ channel antagonist. It interacts with the voltage-activated L-type and T-type Ca^2+^ channels and the slowly gating K (Ca^2+^) channel with varying degrees of specificity and affinity. The binding site is located at the benzothiazepine receptor on the alpha 1-subunit of the channel [8,9]. Recent studies have shown that TET has an inhibitory effect on the efflux pumps in a large range of life forms. It has a reversal effect in P- glycoprotein-mediated drug resistance in cancer cells [10]. It has a synergistic effect on drugs against fungus *Candida albicans*, bacteria methicillin-resistant *Staphylococcus aureus*, and *Mycobacterium smegmatis* [11-13]. Research has showed that efflux pumps across both prokaryotes and eukaryotes are very similar in structures and functions [14]. Strikingly, bacterial efflux pump LmrA was able to functionally complement human efflux pump P-glycoprotein in human lung fibroblast cells [15]. Whatsmore, TET has been shown to have effects paralleling those known calcium antagonists and synthesized efflux pump inhibitors such as verapamil [8]. Based on this amount of information, we can come to a hypothesis that TET may be able to reverse efflux mechanism-mediated drug resistance in MTB. Despite of the relatively large body of literature pointing to this hypothesis, there have not been any experiments to test it. This study shows the effectiveness of TET in the reversal of drug resistance in a group of IEDR MTB clinical isolates.

## Methods

### Bacterial strains and growth conditions

MTB were the clinical isolates from State Key Laboratory for Diagnosis and Treatment of Infectious Diseases (SKL, Hangzhou, Zhejiang, China). H37Rv (Mycobacterium tuberculosis ATCC®25618) was bought from ATCC. The isolates and H37Rv were grown in KaiBiLi modified Lowenstein-Jensen medium (Hangzhou Genesis Biodetection & Biocontrol CO., Ltd., Hangzhou, Zhejiang, China).

### Drugs

TET (potency ≥ 98%) and Rifampicin (97%) were purchased from Aladdin Chemistry Co. Ltd (Shanghai, China). 20 mg TET was dissolved in 800 μl PBS mixed with three drops of hydrochloric acid. Let sit for 5 minutes until transparency appears, then add three drops of 4% sodium hydroxide to neutralize the solution. Dissolved TET was further diluted in Middlebrook 7H9 broth to obtain desired concentration. PBS was made by Hangzhou Ke Yi Biotechnology (Hangzhou, China). INH, EMB and resazurin sodium salt were bought from Sigma-Aldrich (St. Louis, MO, USA). Streptomycin at USP grade was bought from Jing Ke Hong Da Biotechnology (Beijing, China).

### Drug sensitivity assay

Drug sensitivity was measured in 96-well format as previously described and results have been shown to be comparable with those obtained with the conventional proportion method on Lowenstein-Jensen medium [16]. A 100 μl volume of Middlebrook 7H9 broth was dispensed in each well of a 96-well cell culture plate (Corning Incorporated, Corning, NY, USA). Two-fold dilutions of the first-line anti-mycobacterial drugs were directly prepared in the medium. About 1 mg bacteria were put in 800 μl 7H9 broth in a 2-ml test tube containing two 3 mm glass-beads. Bacteria suspensions were prepared by shaking the tube at the highest speed for 1 hour on a shaker from Extractor™ 36 produced by CapitalBio Corporation (Beijing, China). The suspension was further diluted at 1:10 in 7H9 broth. Then inoculate 100 μL diluted bacteria suspension to each well of the plate. Plates were put in a partially zipped plastic bag which has some filter paper soaked in sterile water in it. This is to avoid dehydration of the medium during the incubation. The concentrations of INH after two-fold serial dilution in the medium is 0.0625, 0.125, 0.25, 0.5, 1, 2, 4, 8 μg/ml and that of EMB is 0, 1.25, 2.5, 5, 10, 20, 40, 80 μg/ml. After the incubation at 37°C for 1 week, twenty-five microliters of 0.02% resazurin solution was added to each well. Plates were incubated at 37°C for one additional day. A change in color from blue to pink indicated the growth of bacteria. The minimal inhibitory concentration (MIC) was defined as the minimum drug concentration that prevented the color change in resazurin solution.

### Statistical analysis

The data were analyzed by parametric statistical analysis 1-way ANOVA using OriginPro 8 SR4 PC software, version 8.0 (Micro-Cal; GE Healthcare). Results are expressed as means ± SEM. *P* < 0.05 was considered significant.

## Results

### Selection of IEDR MTB clinical isolates

**Figure 1:**
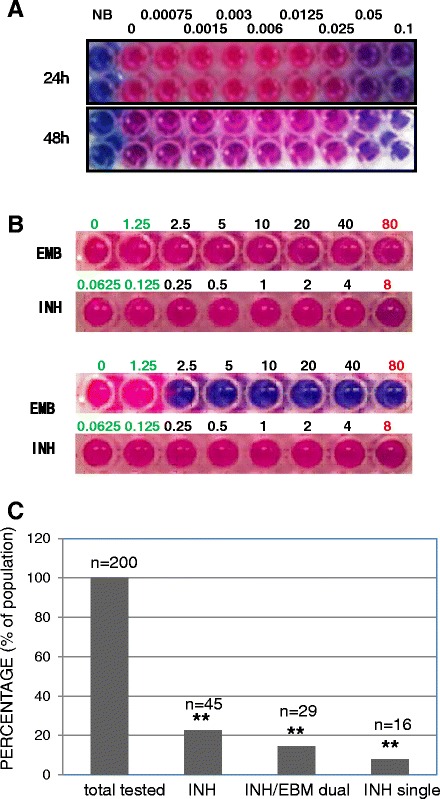
**Screening for IEDR MTB clinical isolates.** 200 MTB isolates from sputum positive culture were subjected to drug sensitivity assay against INH or EMB. The assay used resazurin as oxidation-reduction indicator. A change in color from blue to pink indicated the growth of bacteria, and the MIC was defined as the minimum drug concentration that prevented the color change in resazurin solution. **(A)** H37Rv was tested in drug sensitivity assay. Assay was carried out in duplicates at each concentration of streptomycin. The upper panel shows the 24-hour result and the lower panel shows the 48-hour result. Labels were the concentrations of streptomycin in μg/ml after two-fold serial dilution. **(B)** Upper panel indicates the strain is resistant to both EMB and INH, and the lower panel indicates the strain is resistant to INH but not EMB. Labels were the concentrations of the drug EMB or INH in μg/ml after two-fold serial dilution. Letters in green indicate the normal range of sensitivity. Letters in red indicate clinical level of resistance. **(C)** A statistical result of drug resistance to INH. Two independent experiments were carried out and representative data were shown. **, *p* < 0.01.

Clinical isolates of MTB were collected from several hospitals and local CDCs covering Zhejiang Province, China. These samples should be representative of the MTB circulating in Zhejiang province as studied previously [17,18]. The clinical MTB strains were sputum cultures stored at the State Key Laboratory for Diagnosis and Treatment of Infectious Diseases (SKL, Hangzhou, Zhejiang, China). Growth and morphological characteristics of the colonies were used to exclude non-MTBs. Drug sensitivity assays were carried out on these isolates. The assay was first validated with standard laboratory strain H37Rv carried out in duplicates (Figure 1A). The readout of the duplicated samples under the same concentrations of streptomycin was the same. The readout at the 24-hour time point was similar as the 48-hour time point. The MIC of H37Rv against streptomycin obtained from the drug sensitivity assay was the same as the known data. After the validation of the assay, we moved on to screen for drug-resistant isolates against four first-line anti-tubercular drugs namely INH, EMB, rifampicin and streptomycin. Later we choose to focus on the selection of INH-resistant strains and IEDR strains. The IEDR strains most probably use efflux pumps to generate drug resistance [5,6,19-21]. As shown in Figure 1B, the upper panel showed one isolate resistant to both EMB and INH, whereas the lower panel showed another isolate resistant to INH but susceptible to EMB. Statistical analysis showed that 22.5% of the clinical isolates turned resistant to INH (Figure 1C). This rate was close to the earlier reports of others [17,18]. Interestingly, we observed that over 65% of the INH-resistant isolates harbor resistance to EMB as well (Figure 1C). Our observation is consistent to Gupta’s reports that INH- and EMB-drug resistance often parallel which involves efflux-mediated mechanism [5,6].

### Efflux pump inhibitor verapamil reversed drug resistance of IEDR clinical isolates

**Figure 2:**
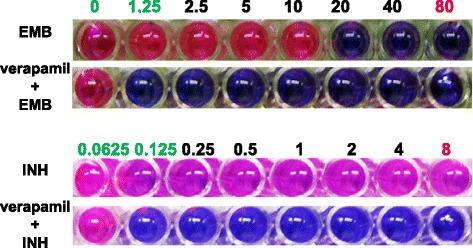
**Verapamil reversed drug resistance in IEDR clinical isolates.** Bacteria were incubated with 5 μg/ml verapamil for 24 hours before anti-tubercular drugs were added. Shown was one strain displaying reversal of the drug resistance to both INH and EMB.

Verapamil is a synthesized efflux pump inhibitor. We first used verapamil to test whether IEDR clinical isolates obtained resistance phenotype via efflux mechanism. If this is the case, combined use of verapamil with the anti-tubercular drugs should be able to minimize the resistance. As shown in Figure 2, the MIC of an IEDR strain, after verapamil treatment, dropped from the level of clinical resistance to normal. The reversal effect of verapamil confirmed that the resistance of IEDR isolates is mediated by efflux pumps. We used all 29 IEDR clinical isolates for verapamil reversal test. The drug resistance was reversed by the combined usage of verapamil in about 50% of tested IEDR isolates. TET appeared more potent than verapamil at the doses used. If verapamil and TET was used together, there might be an additive effect on the reversal effect.

### Effect of TET on the drug killing of IEDR

**Figure 3:**
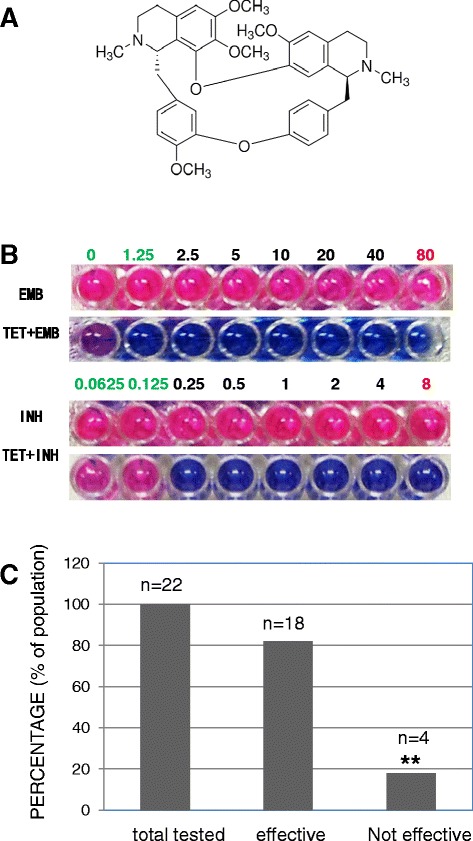
**TET reversed drug resistance in IEDR clinical isolates. (A)** Chemical structure of TET. **(B)** A display of one strain showing that the drug resistance was reversed after TET treatment. Bacteria were incubated with 30 μg/ml TET for 24 hours before anti-tubercular drugs were added. **(C)** A statistical result of TET treatment. Two independent experiments were carried out and representative data were shown. **, *p* < 0.01.

TET is a bis-benzylisoquinoline alkaloid, which is the main active component from the root of *S. tetrandra* S. Moore (Figure 3A). It is known that TET acts as a calcium antagonist similar to verapamil, it is yet not clear if TET is also an efflux pump inhibitor as verapamil [8,22,23]. We next tested whether TET can reverse drug resistance in 29 IEDR strains identified in Figure 1B. We tested the reversal effect of TET on all the isolates and found 18 IEDR isolates were responsive and 4 were non-responders. There are 7 isolates failed to be suspended well and did not meet the assay standard and were excluded from reading. Incubation of the IEDR strains with TET alone did not show that TET had any impact on the growth rate of the strains. As shown in Figure 2B, in one of the strains TET reduced the MIC of EMB by over 64 fold from clinical resistant concentration 80 μg/ml to a sensitive dose 1.25 μg/ml, and it reduced the MIC of INH by over 32 fold from 8 μg/ml to 0.25 μg/ml (Figure 3B). By statistics, 82% of the IEDR strains displayed a reversal effect on the MIC after TET treatment (Figure 3C). These data has shown that TET is an efflux pump inhibitor similar to verapamil. Although others have shown that TET has an antibacterial activity against *Staphylococcus aureus*, as observed in our experiment, TET did not show a direct antibacterial activity against MTB.

## Discussion

In this study, we have for the first time shown that the natural herbal medicine TET played a similar role as the synthesized efflux pump inhibitor verapamil and showed a significant reversal effect on drug resistance in IEDR clinical isolates. Importantly, our data suggest that combined use of TET with drugs such as INH or EMB may be a useful strategy to conquer drug resistance in the majority of patients who were infected by IEDR strains. This application of TET means a lowered drug dose and a shortened duration required in the treatment for these patients.

Efflux pump-mediated drug resistance involves several circumstances, e.g. a result of an increasement in *de novo* synthesis of the pump itself, or activation of a preexisting efflux pump [24]. Permanent up-regulation of efflux-pump expression is also possible. For example, it can occur through mutations in the local repressor gene, in a global regulatory gene/transcriptional activator, in the promoter region of the efflux-pump gene or mutation in insertion elements located upstream of the efflux-pump gene [25]. Recent study has shown that drug tolerance in replicating mycobacteria can arise through a macrophage-induced efflux mechanism, which can persist even after the bacteria leaves the niche [4].

Resistance to INH and EMB often occurs at the same time [5,13,21]. Consistent with an earlier study where more than 85% EMB-resistant isolates showed resistance to INH, we too have found that there is a parallel resistance between EMB and INH in MTB [21]. Our data showed that 65% INH-resistant clinical MTB isolates showed resistance to EMB (Figure 1B). A micro-array study has at the molecular level shown that both INH and EMB were able to induce over-expression of a same set of efflux pump molecules including Rv2459, Rv3728, and Rv3065 [6]. In addition, our study showed that in the presence of an efflux pump inhibitor verapamil, MICs of INH and EMB of the IEDR strains were switched to normal at the same time. These data have shown together that IEDR isolates obtained resistance due to the effect of the efflux pumps. We did the MTB drug resistance detection DNA array test (CapitalBio Corporation, Beijing, China). This test is used to detect mutations in three genes, *i.e. rpoB*, *katG* and *inhA*. In this kit, mutation sites in either *katG* gene or *inhA* gene was tested for the drug resistance against INH. In the 29 IEDR isolates, INH resistance mutations were found in one IEDR isolate that does not respond to TET and five IEDR isolates that did respond to TET. So there appeared no significant genotypic associations of TET responsiveness in these strains. It is possible that drug resistance mediated by different mechanisms co-exists in one strain, but efflux pump may play a more important role [26,27].

Our data showed that TET can reverse drug resistance in 82% of IEDR clinical isolates of MTB (Figure 3C). Because IEDR strains account for about 14.5% of all the 200 clinical isolates tested (Figure 1B), the effective rate of TET among the human population should be 12% minimal. It still remains to further tested whether there are other patterns of drug resistance involving efflux pumps and whether they could too be reversed by TET. Although other patterns remain fully tested, we did observe that TET showed a reversal effect in a few INH single-resistant clinical isolates.

## Conclusions

TET may become a novel drug in combating anti-tubercular drug resistance based on the *in vitro* data we have shown. As is known TET is an existing drug used clinically for different purposes whose safety has been tested and thus would mitigate a significant amount of risk. Developing it as a drug for combined usage to treat tuberculosis would increase the drug efficacy against drug-resistant MTB isolates, and lower the dose administered to alleviate side effects.
